# Dapagliflozin-Induced Acute Pancreatitis: A Case Presentation and Review of the Literature

**DOI:** 10.7759/cureus.62757

**Published:** 2024-06-20

**Authors:** Panagiota Issa, Dimitrios Agapakis, Konstantina Machaira, Karageorgiou Ioannis, Georgios Kotronis

**Affiliations:** 1 Department of Internal Medicine, Aghios Pavlos General Hospital, Thessaloniki, GRC; 2 Diabetes Clinic, Aghios Pavlos General Hospital, Thessaloniki, GRC

**Keywords:** sglt-2 inhibitors, adverse effects, diabetes mellitus, dapagliflozin, drug-induced pancreatitis

## Abstract

Acute pancreatitis has been considered a rare potential adverse effect of sodium-glucose co-transporter-2 inhibitors (SGLT-2is), a new class of medications recently approved for use as an add-on therapy in patients with poorly controlled type 2 diabetes mellitus as well as in individuals with heart failure (HF) and chronic kidney disease (CKD). SGLT-2i can effectively reduce cardiovascular mortality and the deterioration of renal function. There are only a few published cases of acute pancreatitis linked to SGLT-2i administration. Our case describes a 58-year-old male who presented to the emergency department with a clinical presentation of acute pancreatitis, with no known risk factors, who was recently started on therapy with dapagliflozin. Following thorough clinical and laboratory testing, the diagnosis of pancreatitis was associated with dapagliflozin. Upon discharge, dapagliflozin was discontinued with no further recurrence of epigastric pain.

## Introduction

Acute pancreatitis (AP) is a common inflammatory disorder of the pancreas that can vary from mild to severe symptomatology with potentially life-threatening complications. More than 250,000 people with AP are admitted to hospitals every year [1]. The diagnosis of AP is verified in individuals who meet two of the three ensuing criteria: abdominal pain in accordance with AP, serum amylase and/or lipase higher than three times the upper limit of normal, and relevant evidence from abdominal imaging [2]. Long-standing alcohol consumption and biliary stone disease (cholelithiasis) cause the majority of cases of AP [3]; however, there are several other etiologies including post-endoscopic retrograde cholangiopancreatography (ERCP) pancreatitis, hypertriglyceridemia, hypercalcemia, infections, abdominal trauma, autoimmune pancreatitis, hereditary pancreatitis, and surgical procedures and medications [1].

Drug-induced pancreatitis is a relatively uncommon entity (accounting for approximately 2% of all cases) that seems to be linked to an unspecified predisposition. A long list of medications has been associated with AP including azathioprine, sulfonamides, tetracycline, valproic acid, methyldopa, estrogens, furosemide, corticosteroids, and in some cases antidiabetic medications [4].

Furthermore, various hypoglycemic agents have been reported to be linked to AP. While some association exists between the occurrence of pancreatitis and dipeptidyl peptidase 4 inhibitors (DPP4i) and glucagon-like peptide-1 receptor agonists (GLP-1RA) [5], there are only a few reports linking sodium-glucose co-transporter-2 inhibitors (SGLT-2is) with pancreatitis. SGLT-2is have been increasingly used as a treatment for diabetes since the approval of canagliflozin by the Food and Drug Administration (FDA) in 2013. Pancreatitis due to SGLT-2i is a very rare potential adverse drug reaction, with only a few cases reported in the literature.

The following case illustrates a potential dapagliflozin-induced AP.

## Case presentation

A 58-year-old male presented to the emergency department with a three-week history of epigastric pain. He mentioned worsening of the abdominal pain in the last seven days, along with nausea and diarrhea.

On clinical presentation, vital signs included a blood pressure of 150/100 mmHg, a heart rate of 78 bpm, a temperature of 36°C, and an oxygen saturation of 98% on room air. On physical examination, epigastric tenderness with guarding and normoactive bowel sounds was noted. His cardiac and respiratory system evaluation was within normal limits.

The patient's past medical history was significant for type 2 diabetes mellitus (diagnosed five months ago), hypertension, acute coronary syndrome (PCI×2 11 weeks ago), and smoking. He denied any history of cholelithiasis, alcohol abuse, or any prior episode of AP. Medications on a daily basis included clopidogrel, metformin, rosuvastatin, ramipril, rabeprazole, dapagliflozin, manidipine, and torasemide. After thorough examination and questioning, the patient revealed that he was started on dapagliflozin one week prior to the onset of symptoms and noted no other recent changes in his regimen.

Laboratory investigations are shown in Table 1.

**Table 1:** Laboratory investigations during hospitalization WBC: white blood cell; ALT: alanine transaminase; AST: aspartate aminotransferase; LDH: lactate dehydrogenase; ALP: alkaline phosphatase; CRP: C-reactive protein; LDL: low-density lipoprotein; HDL: high-density lipoprotein

Test	Result	Reference range
WBC	9150	4.2-11×10^3^
Neutrophils	70.4%	40-70%
Lymphocytes	17.8%	20-45%
Monocytes	8%	2-11%
Eosinophils	2.6%	0-7%
Blood glucose	88	70-109 mg/dL
ALT	26	-55 IU/L
AST	29	5-34 IU/L
LDH	140	125-243 IU/L
ALP	48	40-150 IU/L
Serum amylase	210	25-125 IU/L
Serum lipase	426	8-78 IU/L
CRP	10.6	-0.5 mg/dL
Triglycerides	158	-150 mg/dL
Total cholesterol	102	-200 mg/dL
LDL cholesterol	56	<70 mg/dL
HDL cholesterol	24	50 mg/dL
IgG1	86	405-1011 mg/dL
IgG2	400	169-786 mg/dL
IgG3	21.50	11-85 mg/dL
IgG4	49.10	3-201 mg/dL
IgG4/IgG ratio	3.6	<6%
Blood ketones	2.3	0.6-1.5 mmol/L

Arterial blood gas values are shown in Table 2.

**Table 2:** Arterial blood gas values on admission pCO2: partial pressure of carbon dioxide; pO2: partial pressure of oxygen;HCO3: bicarbonates; BE: base excess; sO2: oxygen saturation; AnGap: anion gap; Hct: hematocrit; Na: sodium; K: potassium; Cl: chloride; Glu: glucose; Lac: lactates

Test	Result
pH	7.411
pCO2	36.6 mmHg
pO2	76.4 mmHg
HCO3	22.7 mmol/L
BE (ecf)	-1.9 mmol/L
sO2	95.3%
AnGap	21.5 mmol/L
Hct	40%
Na	137 mmol/L
K	3.6 mmol/L
Cl	97 mmol/L
Glu	128 mg/dL
Lac	0.89 mmol/L

**Figure 1:**
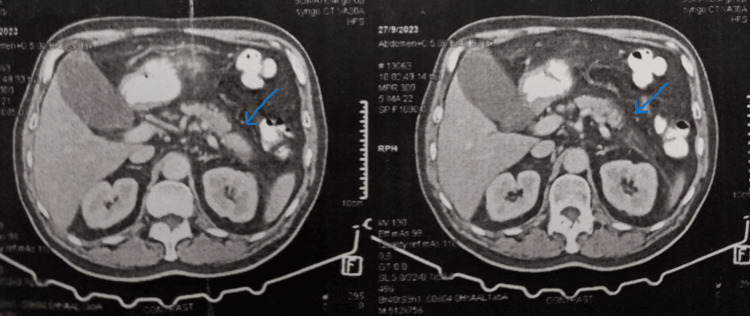
CT scan of the abdomen depicting inflammation of the pancreatic tail CT: computed tomography

Ultrasonography of the upper abdomen didn't depict any pathological findings, while the computed tomography (CT) scan of the abdomen showed mild edema and inflammation of the pancreatic tail, findings consistent with AP (Figure 1).

Further imaging with magnetic resonance cholangiopancreatography (MRCP) did not depict any findings consistent with choledocholithiasis or any abnormality in the pancreatic duct.

The patient was managed with intravenous fluids, analgesics, and insulin and was placed nil per os (NPO). After three days, the patient's symptoms improved and resumed oral food intake with no further regression of the abdominal pain. His amylase and lipase levels returned to normal within five days.

Upon discharge, metformin dosage was increased to twice daily, and dapagliflozin was discontinued.

One-month follow-up visit revealed no further episodes of abdominal pain; however, elevated levels of glucose on daily blood glucose monitoring were noted. Pioglitazone 30 mg daily was added. The patient remained asymptomatic in the three-month follow-up visit.

## Discussion

SGLT-2is are a novel class of oral antidiabetic agents, which lower blood glucose levels by inhibiting SGLT-2, thus decreasing the reabsorption of glucose by the kidney and promoting the excretion of glucose in the urine. When combined with lifestyle modifications such as diet and exercise in adults, these agents assist to improve glycemic control. SGLT-2is may also affect multiple physiological functions including the reduction of both cardiac preload and afterload, attenuation of sympathetic activity, and reduction of intraglomerular pressure [6]. These medications have been investigated both as monotherapy and as an additional treatment with insulin or other oral antidiabetic agents. SGLT-2is are widely used as an add-on therapy in patients with poorly controlled type 2 diabetes mellitus. SGLT-2is have also broad prospects for clinical application and can effectively reduce cardiovascular mortality and the deterioration of renal function. Dapagliflozin and empagliflozin have shown superiority to placebo in reducing heart failure (HF) and renal endpoints regardless of the presence of T2DM; therefore, both have been approved for the management of HF and chronic kidney disease (CKD) [7].

With the broad use of these medications, their safety has drawn increased attention. Some case reports have suggested that SGLT-2is likely lead to AP [8-16]. The majority of these cases pertained to mild to moderate severity of pancreatic inflammation, with the exception of one case in which necrotizing pancreatitis was diagnosed [11]. In 2016, the United States FDA released a hazard signal for the potential association between SGLT-2i AP [17]. In 2018, the Ministry of Health of Canada published the results of SGLT-2i risk evaluation for pancreatitis caused by hypoglycemic agents and suggested that SGLT-2i might be linked to AP [18].

Dapagliflozin, a member of this group of medications, was approved in January 2014 by the FDA for therapeutic use in type 2 diabetes mellitus. Dapagliflozin-induced AP is very rare. Elucidating a mechanism for dapagliflozin-induced AP is challenging. Most cases of drug-induced pancreatitis appear to be idiosyncratic, namely, they have no direct relation to the drug pharmacodynamics [19]. These adverse reactions evolve spontaneously, derive from aberrant interactions between drugs and patients, and are mainly mediated by immunologic or cytotoxic effects provoked by the drug or its derivatives on a specific organ or tissue. It is also unclear whether the level of expression of SGLT-2 in the pancreas could be related to the occurrence of AP.

The first report of dapagliflozin-linked AP dates to 2018. Gutch and Bhattacharya described a 48-year-old patient who developed AP one week after commencing dapagliflozin 10 mg once a day [13]. Thereafter, there have been two similar cases reported in the literature [14,16]. A potentially significant link between the occurrence of AP and the use of SGLT-2is has also been described in an analysis of WHO's global adverse drug reactions database VigiBase® data. This analysis implied a rather class effect, although very rare, that requires further investigation [20].

Nevertheless, the diagnosis of drug-induced pancreatitis, which is a severe, potentially fatal complication, can be easily missed in patients with multiple comorbidities and on multiple medications.

Our patient presented with AP in the setting of newly introduced dapagliflozin in the absence of any known or apparent etiology of pancreatitis. Additionally, no other changes to the patient's medications were reported in the weeks prior to the pancreatitis apart from the commencement of dapagliflozin. Moreover, the inflammatory process was noted in the pancreatic tail, making it unlikely to be associated with any lithiasic event. To add on, no elements of autoimmunity or alcohol abuse were evident or reported. Finally, upon discontinuation of dapagliflozin, the symptoms improved. Based on all the above, the introduction of dapagliflozin stands as the most likely cause of pancreatitis in our patient.

## Conclusions

Clinicians must be well aware of the new antidiabetic medications and their potential side effects and drug interactions, before prescribing any hypoglycemic agent. Drug-induced pancreatitis may be easily overlooked due to its low incidence and concealed by the presence of multiple comorbidities and polypharmacy. Therefore, clinical monitoring during clinical treatment should be strengthened, for proper vigilance of the occurrence of adverse effects like AP.
